# Electric Currents of the Skin

**Published:** 1890-05

**Authors:** 


### ELECTRIC CURRENTS OF THE SKIN.

An interesting study has been lately made by Herr Tarchenoff, of electric currents in the skin from mental excitation. Unpolarizable clay electrodes, connected with a delicate galvanometer, were applied to various parts—hands, fingers, feet, toes, nose, ears and back, and, after compensation of any currents which occurred during rest, the effects of mental stimulation were noted. Light tickling with a brush, causes, after a few seconds' period of latency, a gradually increasing strong deflection. Hot water has a like effect, cold, or the pain from a needle-prick a less. Sound, light, taste and smell stimuli act similarly. If the eyes have been closed some time, mere opening of them causes a considerable deflection from the skin of the hand. It is remarkable that these skin currents also arise when the sensations are merely imagined, Mental effort produces currents varying with its amount. If a person is in tense expectation, the galvanometer mirror makes irregular oscillations. In all the experiments it appeared that, with equal nerve excitation, the strength of the skin currents depended on the degree to which the part of the skin bearing the electrodes was furnished with sweat glands.

